# Factors affecting in-hospital mortality in hypotensive blunt trauma: a retrospective observational study

**DOI:** 10.12701/jyms.2026.43.18

**Published:** 2026-02-10

**Authors:** Jong Min Woo, Sang Won Kim, Su Jeong Shin

**Affiliations:** 1 Department of Emergency Medicine, Yeungnam University College of Medicine, Daegu, Korea; 2 Medical Research Center, Yeungnam University College of Medicine, Daegu, Korea

**Keywords:** Abdominal injuries, Mortality, Shock, Triage

## Abstract

**Background:**

Blunt trauma is a major cause of mortality in the working-age population. Patients who develop hypotension shortly after an injury are at a particularly high risk of death. This nationwide study aimed to identify the factors associated with mortality in patients with hypotension after blunt trauma.

**Methods:**

We analyzed nationwide data from the National Emergency Department Information System for patients aged 15 to 69 years who presented to regional or higher-level emergency medical centers between 2019 and 2023 after blunt trauma. Patients with an initial systolic blood pressure ≤90 mmHg and classified as high acuity (Korean Triage and Acuity Scale ≤3) were included.

**Results:**

Among the 2,713,014 trauma cases, 25,107 met the inclusion criteria, and 16,823 (67.0%) were male. Traffic accidents were the most common reason for injury (38.8%). Mortality was significantly associated with brain injury (hazard ratio, 1.906; 95% confidence interval, 1.661–2.186). The median time from emergency department visit to death was 45.0 hours (interquartile range [IQR], 9.0–188.0 hours), and non-survivors had a median hospital stay of 2.0 days (IQR, 0.0–8.0 days).

**Conclusion:**

Most deaths following blunt trauma occurred within 48 hours of injury, with brain injury being strongly associated with mortality. However, the contribution of other injured body regions may not have been fully captured. These findings underscore the importance of early recognition and comprehensive management of patients with hypotensive blunt trauma.

## Introduction

According to reports from the Centers for Disease Control and Prevention, the burden of unintentional injuries, including drug overdose, traffic accidents, falls, and drowning, has steadily increased over the past decade and represents the fastest growing category among the five leading causes of death [1]. Despite a steadily declining trend, the reported death rate in Korea from preventable trauma continues to vary, ranging from 15.7% to 8.0% [2,3]. A recent follow-up study indicated a reduction to 13.9%; however, this decrease was not statistically significant [4].

The causes of trauma-related mortality vary depending on the study design and patient population; however, hemorrhage (42.7%) and central nervous system injury (27.4%) remain the leading causes of death [4]. An autopsy-based study of traffic accident fatalities indicated that approximately half of the deaths occurred at the scene, with the majority attributed to head injuries [5]. In contrast, a multicenter study focusing on patients with abdominal injuries demonstrated poorer outcomes and longer hospital stays in patients with blunt trauma [6].

A nationwide comparative study using Korean data evaluated mortality outcomes between regional trauma centers and emergency medical centers and reported improved survival in patients with abdominal, pelvic, and lower extremity injuries—injury patterns in which exsanguination is a likely cause of death. The authors attributed this survival advantage primarily to the availability of rapid massive transfusion protocols and timely hemorrhage control interventions, such as angioembolization [7]. An increasing number of investigators agree that the classic trimodal distribution of trauma-related mortality is no longer directly applicable to contemporary trauma care [8,9].

Because trauma epidemiology and outcomes continue to evolve alongside changes in healthcare systems and clinical practice, we conducted an analysis of trauma cases in Korea, focusing specifically on patients with blunt trauma who presented with high initial severity. This study aimed to identify the risk factors for mortality in patients with hypotensive blunt trauma and contribute to efforts to reduce preventable trauma-related deaths.

## Methods

**Ethics statement:** This study was approved by the Institutional Review Board (IRB) of Yeungnam University Hospital (IRB No: 2024-12-038). The requirement for informed consent was waived.

### 1. Study design and setting

**Fig. 1.**
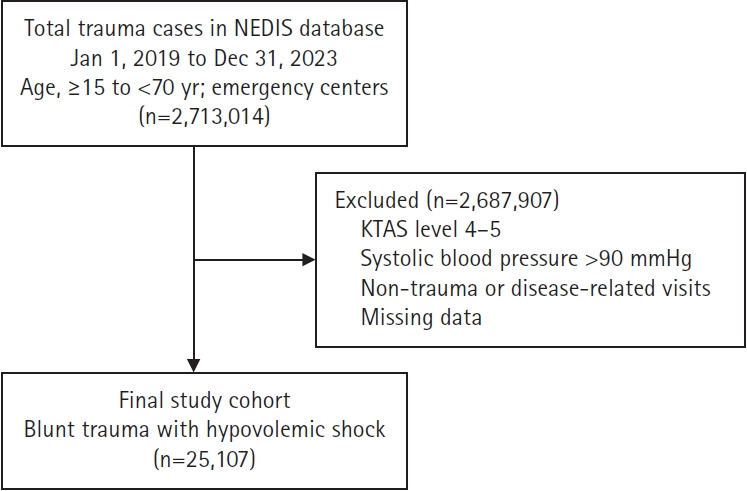
Flow diagram of patient selection. NEDIS, National Emergency Department Information System; KTAS, Korean Triage and Acuity Scale.

We analyzed data from the National Emergency Department Information System (NEDIS), a nationwide emergency care database managed by the National Emergency Medical Center of Korea. The study included patients aged 15 to 69 years who presented to regional or higher-level emergency medical centers between January 1, 2019, and December 31, 2023, following non-disease-related blunt trauma, including traffic accidents, falls, slips, collisions, and machinery-related injuries. Among these patients, those with an initial systolic blood pressure ≤90 mmHg and classified as high acuity (Korean Triage and Acuity Scale [KTAS] level ≤3) on emergency department (ED) arrival were selected for analysis. Patients in whom the primary injured body region involved the extremities were excluded because isolated extremity injuries can be handled by proper splinting with direct compression at the prehospital stage and are thus less likely to be involved in early trauma-related mortality (Fig. 1).

### 2. Data collection and measurements

In Korea, emergency medical institutions are classified into three levels: (1) regional emergency medical centers, (2) local emergency medical centers, and (3) local emergency medical institutions. Each institution is required to transmit patient-level clinical and administrative data to the NEDIS, including the date and time of the ED visit, basic demographic information (such as age and sex), reason for visit (including disease-related or injury-related causes), vital signs at presentation, initial triage acuity level and clinical outcomes, and discharge diagnosis codes [10,11].

Using the data obtained from the NEDIS, we extracted demographic characteristics, including age group and sex, as well as the clinical variables described below for analysis. We included data from regional and local emergency medical centers where detailed information on injured body regions was available. The major injured body regions were classified based on the principal diagnosis recorded at hospital admission, using codes from the Korean Standard Classification of Diseases and Causes of Death, which is based on the International Statistical Classification of Diseases and Related Health Problems.

To assess whether the time of injury during the day influenced patient outcomes, injury time was categorized into 6-hour intervals for analysis. Patients were also stratified according to the injury mechanism, route of presentation to the ED, and type of emergency medical institution. Time-related variables were categorized based on the recorded time of injury, which was defined as the incident occurrence time transmitted to the NEDIS. The time intervals from injury to surgery and from injury to hospital admission or death were reported in hours, whereas the length of hospital stay was reported in days, owing to wide variability.

For all the variables described above, patients were classified into survivor and non-survivor groups based on their final clinical outcomes. Comparative analyses between the two groups were performed, and the mortality risk of the non-survivor group versus that of the survivor group was evaluated.

### 3. Statistical analysis

The general characteristics of the study population are summarized using frequencies and percentages. Differences between survivors and non-survivors were compared using the chi-square test or Fisher exact test, as appropriate. Continuous variables, including time to operating room transfer and ED length of stay, were assessed for normality and are presented as medians with interquartile ranges (IQRs). Comparisons between groups were performed using the Wilcoxon rank-sum test (Mann-Whitney U-test).

A Cox proportional hazards model was used to identify factors associated with mortality and to estimate hazard ratios (HRs) with 95% confidence intervals (CIs); the proportional hazards assumption was evaluated using log–log survival plots. All statistical analyses were conducted using SAS software, version 9.4 (2023; SAS Institute, Cary, NC, USA). A two-sided *p*-value <0.05 was considered statistically significant.

## Results

### 1. Patient general characteristics

A total of 25,107 patients were included in the study. The percentage of patients sustaining hypotensive blunt trauma increased with advancing age in both the survivor and non-survivor groups. Male patients comprised the majority of the cohort (16,823 patients; 67.0%). Although lower KTAS levels, indicating higher initial severity, were less frequent overall, the opposite distribution was observed in the non-survivor group, in which a higher proportion of patients presented with lower KTAS levels.

Analysis of the major injured body regions showed that head injury was the most common injury across all groups (7,218 patients; 28.7%). Injuries occurred more frequently in the afternoon and evening; however, this temporal pattern was less consistent in the non-survivor group. Traffic accidents were the most common cause of injury in both survivors and non-survivors. Most patients (21,150; 84.2%) visited emergency medical centers directly; among non-survivors, a high proportion visited regional emergency medical centers (1,374; 69.5%). The median time from ED arrival to surgery was 4.0 hours (IQR, 3.0–5.0 hours) in the non-survivor group and 5.0 hours (IQR, 3.0–7.0 hours) in the survivor group. The median time to death was 45.0 hours (IQR, 9.0–188.0 hours), while the median length of hospital stay among survivors was 17.0 days (IQR, 8.0–34.0 days) (Table 1).

### 2. Multivariate analysis

In the multivariate analysis of factors associated with mortality, increasing age was significantly associated with a higher risk of death, especially in the >50-year age group. Compared with younger patients, those aged 51 to 60 years (HR, 1.498; 95% CI, 1.106–2.029) and 61 to 69 years (HR, 2.007; 95% CI, 1.492–2.699) had a significantly increased risk of mortality. Male sex was also associated with a higher risk of death than female sex (HR, 1.264; 95% CI, 1.101–1.450). With respect to initial severity, KTAS level 1 (HR, 19.024; 95% CI, 14.617–24.760) and KTAS level 2 (HR, 4.762; 95% CI, 3.614–6.275) were associated with a significantly higher mortality risk than KTAS level 3.

Head injuries (HR, 1.906; 95% CI, 1.661–2.186) were associated with a significantly higher mortality risk than abdominal and pelvic injuries. Neck injury was associated with lower mortality (HR, 0.730; 95% CI, 0.543–0.981), whereas chest injury was not (HR, 0.933; 95% CI, 0.771–1.129). Regarding the type of medical institution, compared with local emergency medical centers, presentations to regional emergency medical centers were associated with a significantly higher risk of mortality (HR, 1.296; 95% CI, 1.148–1.463) (Table 2).

## Discussion

This study aimed to identify the factors influencing mortality among patients with hypotensive blunt trauma by analyzing nationwide data collected over a 5-year period. Although multiple factors contributed to trauma-related mortality, the higher mortality observed among patients with greater initial severity, as reflected by lower triage levels, may be attributable, at least in part, to the inherent limitations of prehospital trauma care. While various countries and trauma centers have developed and implemented their own triage systems, common challenges have been reported across these systems, including limited sensitivity and the risk of undertriage [12,13]. Strategies such as repeated ultrasonography and pan-scan computed tomography have been increasingly adopted to compensate for the limitations of the existing triage systems and ensure timely and appropriate diagnosis and treatment [14,15].

Numerous studies have emphasized that the risk of mortality increases with advancing age at the time of injury. Especially in individuals aged 70 years and older, even low-height falls from standing level can result in severe injuries and death [9,12,16,17]. Among older individuals, the risk of mortality is higher, even with comparable injury severity, owing to age-related declines in sensory and motor functions, the presence of multiple comorbidities, polypharmacy, and, particularly in women, an increased risk of fractures associated with osteoporosis. Consequently, some guidelines recommend incorporating comprehensive medical management by geriatric specialists into the care of older patients with trauma [18-20]. Because this study focused on patients aged <70 years to better characterize outcomes in the working-age population, the elevated mortality risk observed in patients in their fifth and sixth decades should be interpreted within this contextual framework.

A higher proportion of males has been consistently reported across various types of blunt trauma. This finding has often been attributed to greater levels of social and occupational activity among men. However, further comparative studies across different social and structural contexts are warranted [5,9,21,22]. A trend toward increasing trauma incidence during the afternoon and evening hours has also been reported in international studies [23], which may be related to higher levels of human activity during these periods. Traffic accidents were consistently reported as the most common mechanism of blunt trauma, a finding that is consistent with previous studies [6,9].

Large-scale studies have consistently reported that traumatic brain injury is associated with the highest mortality rate among all trauma-related causes. A Delphi study on preventable trauma-related deaths indicated that outcomes of traumatic brain injury often do not correlate well with conventional severity scoring systems such as the Injury Severity Score and the Trauma Score and Injury Severity Score [24]. Reflecting on these limitations, several studies on blunt trauma have excluded patients with brain injury from their analyses [25]. The findings from one study suggested that the risk of blunt cerebrovascular injury from low-energy mechanisms such as falls is not substantially different from that observed in high-energy trauma, underscoring the need for heightened vigilance regarding the potential coexistence of cervical spine injuries [26]. An autopsy-based study conducted in Canada on patients who died following blunt trauma indicated that abdominal injuries, including liver and splenic ruptures and mesenteric injuries, were more prevalent than head injuries. Notably, the same study found a 100% rate of diagnostic discrepancies in cases that resulted in ED death [27].

With respect to injured body regions associated with mortality, head injuries accounted for a substantial proportion of deaths in this cohort. However, in the ED setting, it is often not feasible to accurately determine the direct cause of death in all patients. Although head injury is not typically a direct cause of hypotension, concomitant hemorrhagic injuries involving other body regions may coexist and contribute to fatal outcomes. Therefore, the observed association between specific injured body regions and mortality should be interpreted with caution. In addition, longer preoperative time intervals were observed among non-survivors; however, causal inferences could not be drawn from these findings. These time-related observations likely reflect the complexity and severity of the injury, rather than a direct effect of treatment delay.

This study had several limitations. First, the major injured body regions were determined solely based on the principal diagnosis at admission, which limited our ability to account for multiple injured regions in individual patients. Consequently, the contribution of injuries in other body regions to mortality may have been underestimated. Therefore, unrecognized injuries in other body regions may not have been fully captured in the analysis and could have contributed to mortality. Generally, head injury itself is not considered to be a direct cause of hypotension, which is closely related to the high severity of trauma in patients with multiple injuries and involves mechanisms such as secondary hypoxia and decompensated metabolic acidosis.

Second, because the NEDIS does not track patients across institutions, transferred patients treated at multiple hospitals may have been counted more than once. This limitation also introduces a potential bias in the time-dependent analyses, including time to operation and time to death, as these intervals may be influenced by transfer-related delays and the selection of patients requiring higher-level care. In addition, because Regional Trauma Centers in Korea operate independent databases that are not fully integrated into the NEDIS, patients treated at these centers were not included in the present analysis. As a result, the findings primarily reflect outcomes from regional emergency medical centers and may not be generalizable to all trauma care settings. To address these limitations, future analyses restricted to patients with direct ED presentation or analyses that link individual patients across institutions may help reduce transfer-related bias and better characterize the impact of interhospital transfer on time-dependent outcomes.

In this nationwide study, advanced age, male sex, higher initial severity, and head injury were associated with increased mortality in patients with hypotensive blunt trauma. As most deaths occurred within 48 hours of injury, early identification of patients who are at high risk and timely system-level interventions are critical to reducing preventable trauma-related mortality.

## Figures and Tables

**Table 1.** General characteristics of patients with hypotensive blunt trauma

Characteristic	Total	Non-survivor group	Survivor group
No. of patients	25,107	1,978	23,129
Age (yr)			
15–20	1,687	77 (4.6)	1,610 (95.4)
21–30	3,423	192 (5.6)	3,231 (94.4)
31–40	2,993	147 (4.9)	2,846 (95.1)
41–50	3,888	278 (7.2)	3,610 (92.8)
51–60	6,108	523 (8.6)	5,585 (91.4)
61–69	7,008	761 (10.9)	6,247 (89.1)
Male sex	16,823	1,553 (9.2)	15,270 (90.8)
KTAS			
1	4,161	1,345 (32.3)	2,816 (67.7)
2	6,435	500 (7.8)	5,935 (92.2)
3	14,511	133 (0.9)	14,378 (99.1)
Major injured part			
Head	7,218	675 (9.4)	6,543 (90.6)
Neck	735	52 (7.1)	683 (92.9)
Chest	2,428	164 (6.8)	2,264 (93.2)
Abdomen and pelvis	3,510	307 (8.7)	3,203 (91.3)
Time of injury (hr)			
00:00–05:59	4,245	330 (7.8)	3,915 (92.2)
06:00–11:59	5,987	578 (9.7)	5,409 (90.3)
12:00–17:59	7,419	584 (7.9)	6,835 (92.1)
18:00–23:59	7,456	486 (6.5)	6,970 (93.5)
Mechanism of trauma			
Traffic accident	9,735	1,070 (11.1)	8,665 (88.9)
Fall from height	5,145	544 (10.6)	4,601 (89.4)
Slip-down	5,908	141 (2.4)	5,767 (97.6)
Others	4,319	216 (5.0)	4,103 (95.0)
Mode of visit			
Direct	21,150	1,511 (7.1)	19,639 (92.9)
Transfer	3,872	466 (12.0)	3,406 (88.0)
Others	85	1 (1.2)	84 (98.8)
Level of the center			
Regional EMC	13,485	1,374 (10.2)	12,111 (89.8)
Local EMC	11,622	604 (5.2)	11,018 (94.8)
Time to OR (hr)	3,409	4.0 (3.0–5.0)	5.0 (3.0–7.0)
Time to admission (hr)	25,046	1.0 (0.0–2.0)	1.0 (0.0–2.0)
Time to death (hr)	1,972	45.0 (9.0–188.0)	-
Hospital day (day)	12,631	2.0 (0.0–8.0)	17.0 (8.0–34.0)

Values are presented as number only, number (%), or median (interquartile range). KTAS, Korean Triage and Acuity Scale; EMC, emergency medical center; OR, operating room. All *p*-values were <0.001.

**Table 2.** Risk factors for mortality in patients with hypotensive blunt trauma

Variable	Hazard ratio (95% CI)	
	Univariate	Multivariate
Age (yr)^a)^		
21–30	1.059 (0.812–1.381)	1.248 (0.895–1.740)
31–40	0.843 (0.639–1.112)	1.108 (0.783–1.568)
41–50	1.071 (0.831–1.381)	1.321 (0.960–1.819)
51–60	1.203 (0.946–1.531)	1.498 (1.106–2.029)
61–69	1.394 (1.101–1.765)	2.007 (1.492–2.699)
Male sex	1.511 (1.357–1.683)	1.264 (1.101–1.450)
KTAS^b)^		
1	18.807 (15.733–22.482)	19.024 (14.617–24.760)
2	3.800 (3.137–4.602)	4.762 (3.614–6.275)
Major injured part^c)^		
Head	2.365 (2.065–2.708)	1.906 (1.661–2.186)
Neck	0.869 (0.647–1.166)	0.730 (0.543–0.981)
Chest	0.981 (0.811–1.188)	0.933 (0.771–1.129)
Level of the center^d)^		
Regional EMC	1.597 (1.451–1.758)	1.296 (1.148–1.463)

CI, confidence interval; KTAS, Korean Triage and Acuity Scale; EMC, emergency medical center. References: ^a)^15–20 years; ^b)^KTAS 3; ^c)^abdomen and pelvis; and d)local EMC.
